# FoxR2 promotes glioma proliferation by suppression of the p27 pathway

**DOI:** 10.18632/oncotarget.17447

**Published:** 2017-04-27

**Authors:** Xuejiao Liu, Ning Liu, Chenglong Yue, Dacheng Wang, Zhenglei Qi, Yiming Tu, Guokun Zhuang, Di Zhou, Shangfeng Gao, Mingshan Niu, Rutong Yu

**Affiliations:** ^1^ Insititute of Nervous System Diseases, Xuzhou Medical University, Xuzhou, Jiangsu, China; ^2^ Brain Hospital, The Affiliated Hospital of Xuzhou Medical University, Xuzhou, Jiangsu, China; ^3^ Jiangsu Key Laboratory of Bone Marrow Stem Cell, Xuzhou Medical University, Xuzhou, Jiangsu, China; ^4^ Department of Neurosurgery, Huzhou Central Hospital, Huzhou, Zhejiang, China

**Keywords:** glioma, FoxR2, proliferation, migration, p27

## Abstract

FoxR2 plays an important role in the development of many human tumors. However, the effects of FoxR2 on tumorigenicity of human glioma remain unclear. In this study, we investigated the roles of FoxR2 in cell proliferation and invasion of glioma. We found that overexpression of FoxR2 promoted the proliferation, migration and invasion of glioma cells. Knockout of FoxR2 induced G1 arrest by decreasing the expression levels of cyclin D1, cyclin E and p-Rb. Mechanistically, upregulation of FoxR2 increased the level and activity of MMP-2 and decreased the expression of p27. Furthermore, overexpression of FoxR2 decreased the nuclear accumulation of p27. Taken together, these results indicate that upregulation of FoxR2 may confer enhanced tumorigenicity in glioma cells.

## INTRODUCTION

Malignant gliomas are the most frequent and aggressive central nervous system tumors. Glioblastoma multiforme (GBM) accounts for approximately 60 to 70% of malignant gliomas [1]. Despite advances in surgery and adjuvant therapy, the median survival is merely 12 to 15 months for patients with GBM [2]. Thus, there is an urgent need to understand biological signaling mechanisms of cell proliferation and invasion in gliomas.

Human *Forkhead-box (Fox)* genes belong to the family of winged/forkhead transcription factors, including at least 43 members from *FoxA1* to *FoxQ1* [3]. Deregulation of Fox family genes could contribute to glioma proliferation and development [4]. For example, FoxM1 is overexpressed in human GBM and contributes to the tumorigenicity of glioma [5]. FoxO3a is a critical regulator of cellular signal pathways and controls the differentiation and tumorigenicity of GBM stem-like cells [6]. The expression of FoxP3 in glioma cells is significantly enhanced after exposure to chemotherapeutics, which induces significant cell apoptosis [7]. These data suggest that the Fox families may serve as potential therapeutic targets for human malignant gliomas.

FoxR2, a new member of Fox transcription factor family, was first identified in 2004 [8]. Recently, FoxR2 has been identified as a potential oncogene in malignant peripheral nerve sheath tumors and medulloblastoma through genome-wide functional screens [9, 10]. FoxR2 is overexpressed in breast cancer cells and associated with poor prognosis [11, 12]. FoxR2 is also high expressed in human hepatocellular carcinoma and promotes proliferation of tumor cells [13]. Recently, it has been demonstrated that FoxR2 could act with Myc to promote tumor cell proliferation [14]. However, the roles of FoxR2 in human glioma development remain unknown.

In this study, we investigated the roles of FoxR2 in the tumorigenicity of glioma. We provided evidence that FoxR2 promotes glioma cell proliferation, migration and invasion through regulating the expression of p27 and MMP-2. Our study provides insights into the applicability of using the FoxR2 as a potential therapeutic target in gliomas.

## RESULTS

### FoxR2 is expressed in human glioma tissues

**Figure 1:**
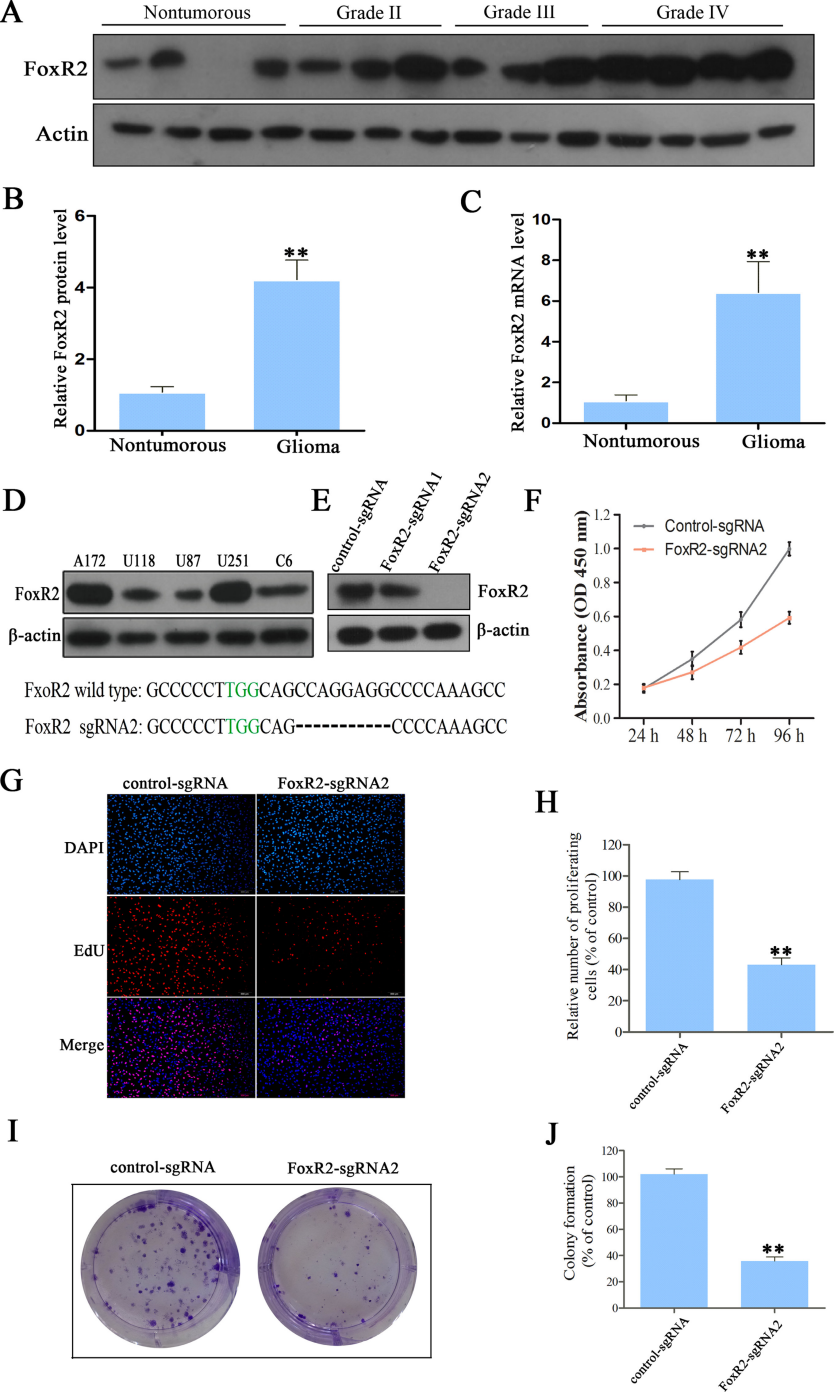
The effects of FoxR2 knockout on cell proliferation of glioma (**A**) Representative protein level of FoxR2 in nontumorous brain tissues and glioma tissues. (**B**) Statistical analysis of the relative protein level of FoxR2 in nontumorous brain tissues (*n* = 9) and glioma tissues (*n* = 33), **P < 0.01. (**C**) The relative FoxR2 mRNA expression in nontumorous brain tissues and glioma tissues as measured by real time RT-PCR. For each sample, the relative FoxR2 mRNA level was normalized to that of GAPDH, **P < 0.01. (**D**) The expression analysis of FoxR2 in five glioma cell lines by western blot analysis. (**E**) Knockout efficiency of FoxR2 was examined by western blot analysis in FoxR2-sgRNA1, FoxR2-sgRNA2 and corresponding control cells. (**F**) CCK8 assay was used to detect the cell viability in control-sgRNA and FoxR2-sgRNA1 U251 cells. (**G**) The proliferative abilities of FoxR2 knockout cells were assessed by the EdU incorporation assay. Representative images of EdU (red) and DAPI (blue) are showed, scale bar: 200 μm. (**H**) Quantification of the percentage of EdU-positive cells. (**I**) Cell proliferation ability after FoxR2 knockout was examined by colony formation assay. (**J**) Quantitative results of colony formation assay. The percentage of proliferative cells and the amount of colony formation were normalized to the corresponding values of the control-sgRNA group. All the results were presented as the mean ± SEM from 3 independent experiments, *P < 0.05, **P < 0.01.

In order to investigate the potential roles of FoxR2 in the development of glioma, we first assessed the protein and mRNA levels of FoxR2 in clinical glioma samples and non-tumorous brain tissues by Western blot and real-time RT-PCR, respectively. As shown in Figure 1A and 1B, the human glioma tissue specimens apparently had a higher level of FoxR2 expression than non-tumorous tissues. Real-time RT-PCR analysis showed that mRNA levels of FoxR2 were also high expressed in glioma samples (Figure 1C). Furthermore, glioma patient samples harbored FoxR2 copy number amplification (4%) and missense mutations (1.8%) by analysis of COSMIC online database. These results indicate that FoxR2 may play a role in the tumorigenicity of glioma.

### Overexpression of FoxR2 promotes proliferation of glioma cells

To determine whether FoxR2 plays an important role in the pathogenesis of glioma, we generated FoxR2 knockout or overexpression glioma cells. We examined the protein levels of FoxR2 in five glioma cell lines using Western blot analysis. As shown in Figure 1D, FoxR2 was overexpressed in U251 and A172 cells, while the expression of endogenous FoxR2 was relative low in U87 and U118 cells. Thus, we chose U251 cells to perform the FoxR2 knockout and U87 cells to overexpress FoxR2, respectively.

The knockout efficiency of FoxR2-sgRNAs was confirmed by western blot analysis. As shown in Figure 1E, FoxR2 was effectively knocked out by FoxR2-sgRNA2. To elucidate the effects of FoxR2 on cell proliferation, we preformed CCK-8, EdU and colony formation assays. Compared with the control-sgRNA group, the cell numbers decreased by 48.5% in FoxR2-sgRNA2 group after incubation for 96 h (Figure 1F). The EdU assay showed that the mean percentage of positive proliferative cells of FoxR2-sgRNA2 group reduced by 51.32 % (Figure 1G and 1H). Furthermore, the colony formation assay indicated that knockout of FoxR2 significantly decreased the ability in colony formation (Figure 1I and 1J).

**Figure 2:**
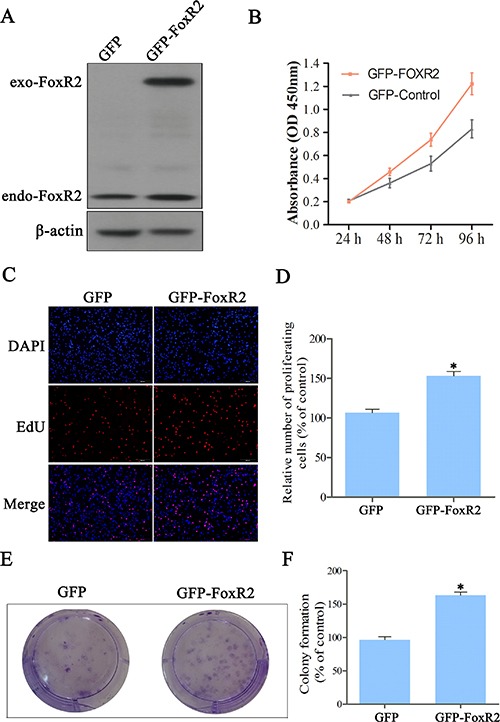
Overexpression of FoxR2 promotes proliferation of glioma cells (**A**) The efficiency of FoxR2 overexpression in U87 cells. Total protein extracts were evaluated through western blot analysis. (**B**) The viabilities of FoxR2 overexpression cells were evaluated by CCK8 assay. (**C**) Representative images from EdU analysis of cell proliferation. (**D**) Quantitative results of EdU incorporation assay. (**E**) Colony formation ability was assessed. (**F**) Quantitative results of colony formation assay. The percentage of proliferative cells and the amount of colony formation were normalized to the corresponding values of the control-sgRNA group. All the data were expressed as the mean ± SEM from three independent experiments, *P < 0.05, **P < 0.01.

Next, we investigated the effects of overexpression FoxR2 on glioma cell proliferation. We transfected U87 cells with the FoxR2 expression lentivirus vector. The efficiency of FoxR2 overexpression was assessed by western blot analysis (Figure 2A). Compared with the control group, overexpression of FoxR2 significantly increased the growth of U87 cells (Figure 2B). As shown in Figure 2C and 2D, the mean percentage of EdU positive cells increased by 50% in GFP-FoxR2 group compared with control cells. Furthermore, overexpression of FoxR2 increased the colony formation ability (Figure 2E and 2F). Taken together, these data demonstrate that FoxR2 can effectively promote the proliferation of glioma cells.

### Knockout of FoxR2 induces G_1_ cell cycle arrest in glioma cells

**Figure 3:**
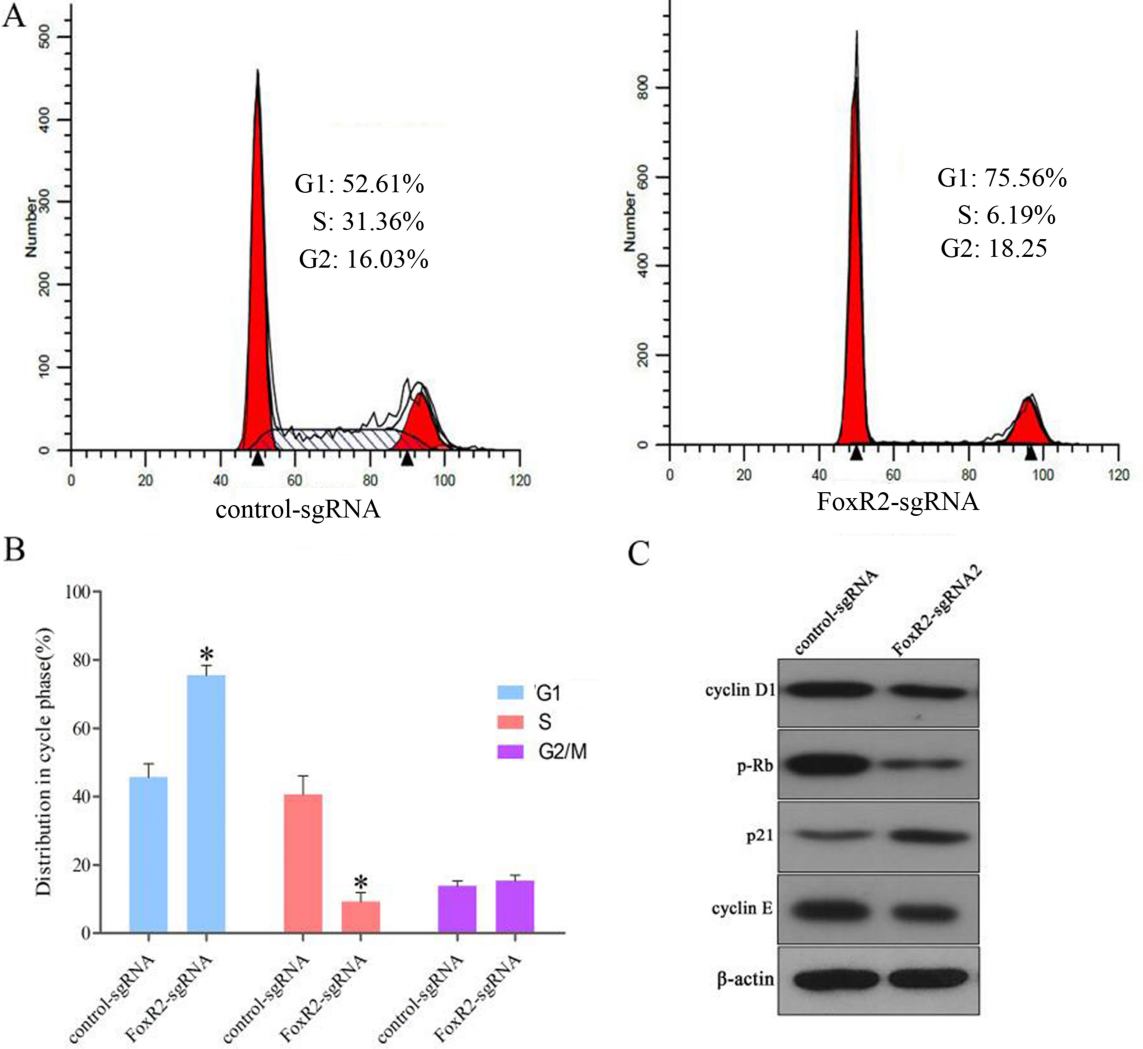
FoxR2 knockout induces cell cycle arrest and modulates expression of cell cycle regulators (**A**) The distribution of cell cycle after FoxR2 knockout was examined by flow cytometry in U251 cells. (**B**) Quantitative analysis of cell cycle phase distribution in the control-sgRNA group and the FoxR2-sgRNA2 group. Triplicate experiments were performed, (**P < 0.01). (**C**) FoxR2 knockout regulates the expression of cell cycle-related proteins.

To further examine the effects of FoxR2 on glioma cell proliferation, we used flow cytometry assays to evaluate cell cycle distribution. In the control-sgRNA cells, 52% of cells were in the G1 phase, whereas FoxR2-knockout cells exhibited a higher percentages of cells (75%) in the G1 phase (Figure 3A and 3B). Conversely, the percentage of FoxR2-knockout cells in S phase was significantly decreased relative to control group cells. These data indicate that knockout of FoxR2 expression inhibits cell cycle progression of glioma cells.

We next examined the effect of FoxR2 knockout on cell cycle regulatory protein by western blot analysis. As shown in Figure 3C, knockout of FoxR2 significantly up-regulated the expression level of cell cycle inhibitory protein p21. In addition, the expression levels of cyclin D1, cyclin E and p-Rb were markedly decreased in FoxR2-knockout cells compared with control cells. These results suggest that FoxR2 modulates multiple cell cycle regulatory gene in glioma cells.

### FoxR2 promotes migration and invasion of glioma cells

**Figure 4:**
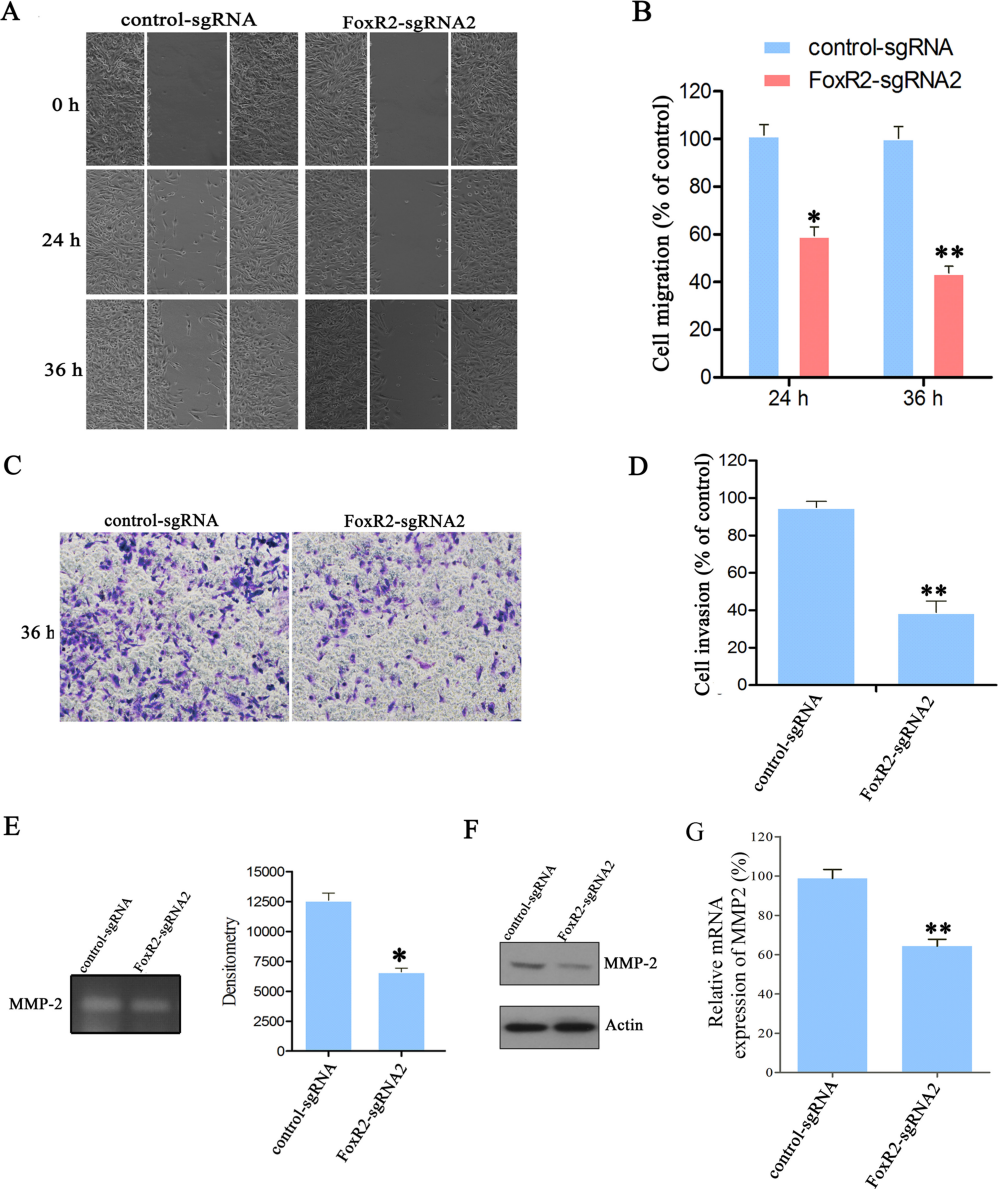
Knockout of FoxR2 inhibits the migration and invasion of glioma cells (**A**) Cell migratory ability of different groups were detected by wound healing assay in U251 cells. (**B**) Quantitative analysis of migratory cell numbers. (**C**) Cell migratory ability after FoxR2 knockout was assessed by transwell invasion assay. (**D**) Quantitative cell numbers invaded through the filter. The numbers of migratory or invading cells were normalized to the control-sgRNA group. (**E**) Representative picture and quantitative MMP-2 activity of gelatin zymography assay. FoxR2-sgRNA2 and control cells were starved in serum-free DMEM for 24 h, then gelatin zymography assay was performed. (**F**) The effects of FoxR2 knockout on MMP-2 protein levels by western blot analysis. (**G**) The effects of FoxR2 knockout on mRNA levels of MMP-2 by Real time PCR analysis. The above results are expressed as the mean ± SEM from three independent experiments, *P < 0.05, **P < 0.01.

**Figure 5:**
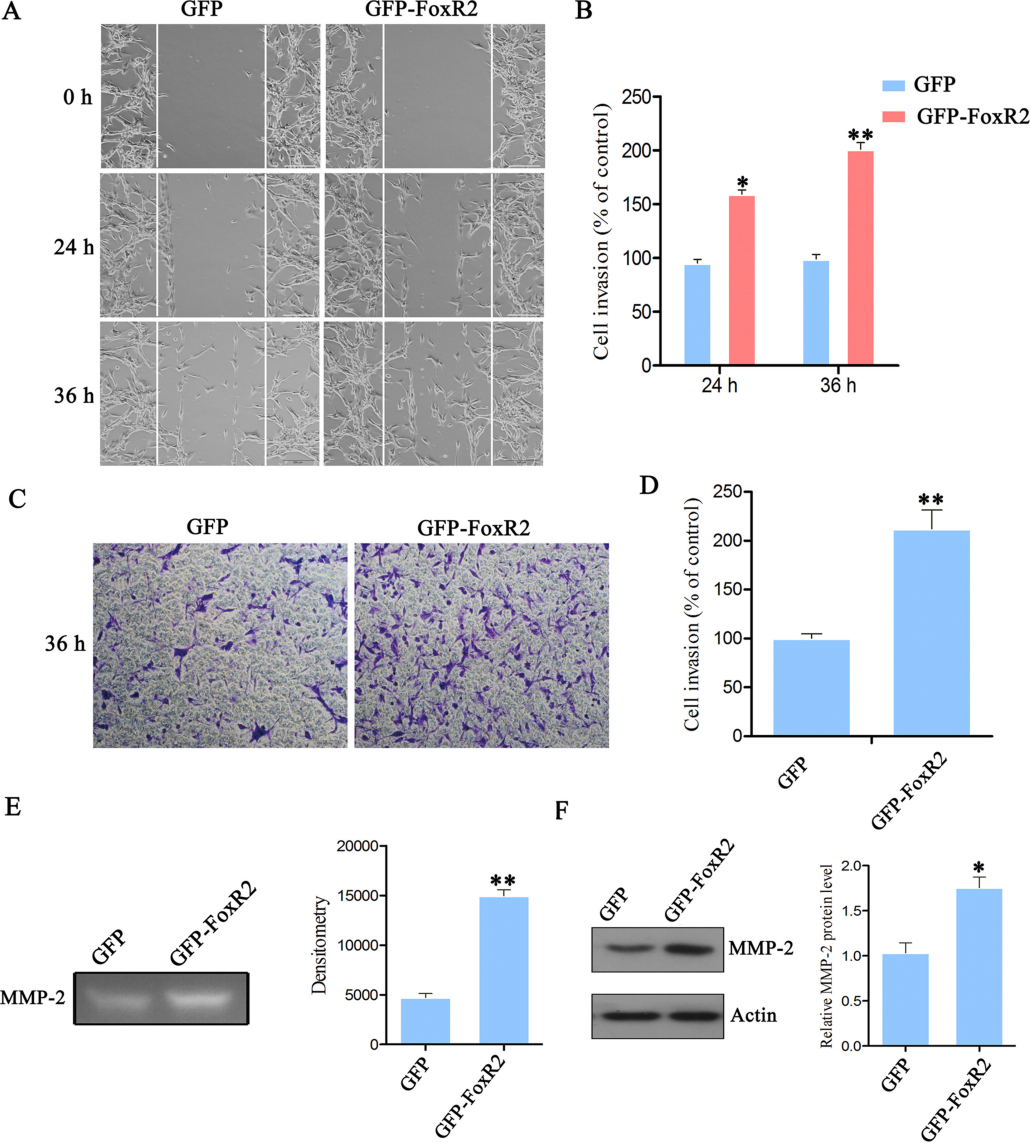
Overexpression of FoxR2 promotes the migration and invasion of glioma cells (**A**) Wound healing assay was utilized to assess cell migratory ability after FoxR2 overexpression in U87 cells. Representative images were taken at 0, 24 and 36 h after scratching. (**B**) Quantitative analysis of migratory cell numbers. (**C**) Transwell invasion assay was performed in GFP and GFP-FoxR2 cells. (**D**) Quantitative analysis of invading cell numbers. The numbers of migratory or invading cells were normalized to that of the control group. (**E**) Gelatin zymography assay was performed to evaluate the effect of FoxR2 overexpression on MMP-2 activity. (**F**) The effects of FoxR2 overexpression on MMP-2 protein levels by western blot analysis. The above results are expressed as the mean ± SEM from three independent experiments, *P < 0.05, **P < 0.01.

To assess the role of FoxR2 in migration and invasion of glioma cells, wound healing and transwell invasion assays were performed. Compared with control-sgRNA group, the migratory cell numbers in FoxR2-sgRNA2 group were reduced by 56.15% after incubation for 36 h (Figure 4A and 4B). The transwell invasion assay showed that the percentage of invasive cells decreased approximately by 60% in FoxR2 knockout group compared with control cells (Figure 4C and 4D). Next, we performed wound healing and transwell invasion assays in FoxR2-overexpressed glioma cells. Compared with control group, the migratory cell numbers of FoxR2 overexpression group increased to 200% after incubation for 36 h (Figure 5A and 5B). Furthermore, the invasive cell numbers increased to 220% upon overexpression of FoxR2 (Figure 5C and 5D). In conclusion, these results suggest that FoxR2 is involved in the migration and invasion function of glioma cells.

MMP-2 can degrade ECM molecules, thereby facilitating cancer cell invasion. The activity of MMP-2 is considered as an index of tumor invasion ability [15, 16]. Thus, we examined the effects of FoxR2 on activity and expression of MMP-2 by gelatin zymography assay and western blot analysis, respectively. As shown in Figure 4E–4G, the excretion and expression levels of MMP-2 were decreased significantly after FoxR2 knockout. In contrast, overexpression of FoxR2 promoted the excretion of MMP-2 and upregulated the expression levels of MMP-2 (Figure 5E–5F). These data indicate that FoxR2 may promote the activity of MMP-2 by increasing its expression level.

### FoxR2 decreases the expression of p27 in glioma cells

**Figure 6:**
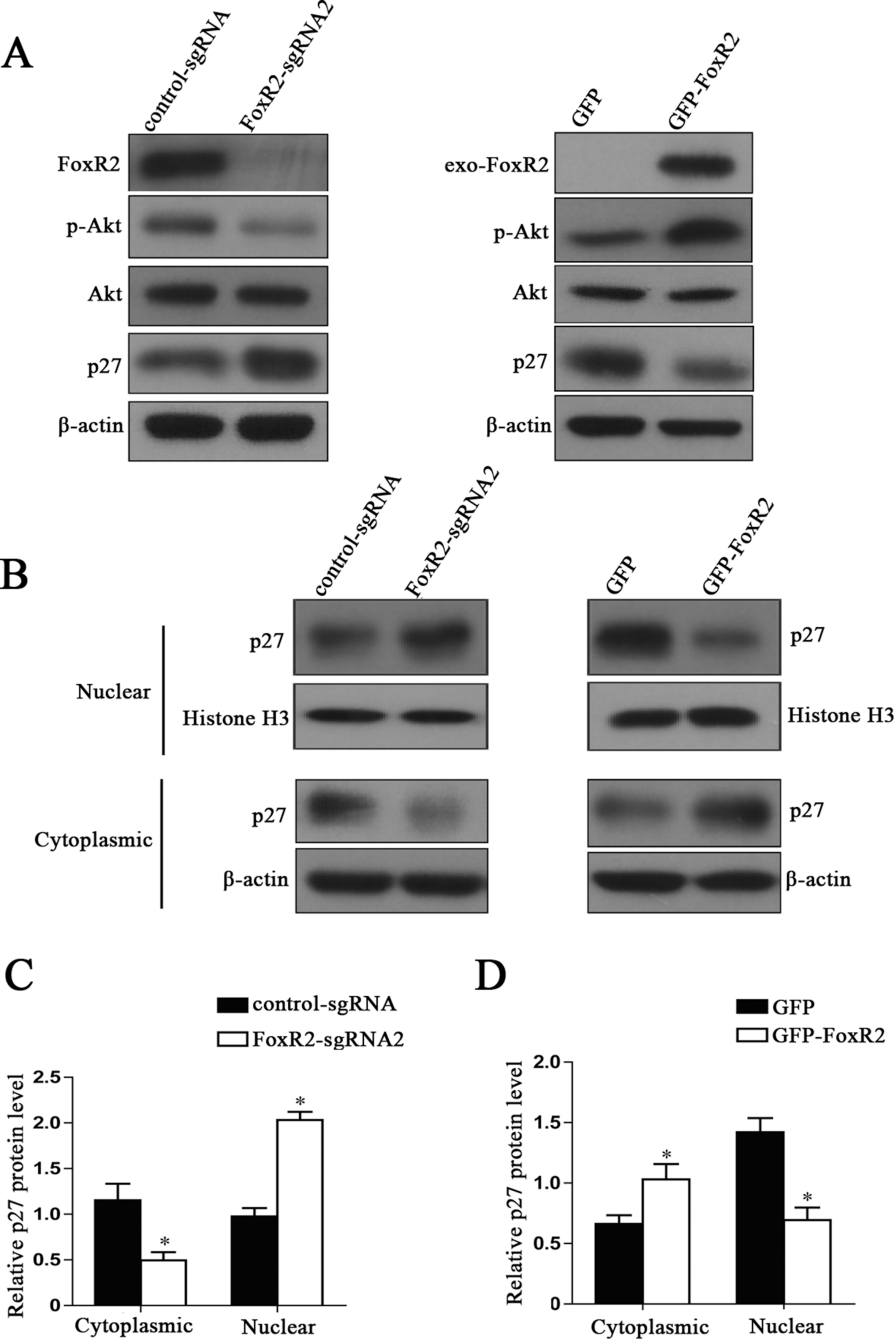
FoxR2 regulates the expression and subcellular location of p27 (**A**) The effects of FoxR2 on the levels of total Akt, p-Akt and p27 by western blot analysis. (**B**) The effects of FoxR2 on the subcellular location of p27. The cytoplasmic and nuclear protein extracts were used for immunoblotting with the indicated antibodies. (**C** and **D**) Quantitative analysis of relative levels of p27 in cell cytoplasm and nucleus, *P < 0.05.

It is well known that p27 is a tumor suppressor gene, which contributes to negatively regulate cell cycle progression in the nucleus [17]. Recntly, it has been demonstrated that cytoplasmic p27 is oncogenic [18, 19]. Akt can phosphorylate p27, which results in cytoplasmic localization of p27 [20]. Furthermore, PI3K/Akt/p27 pathway plays important roles in cell migration and invasion [21–23]. As shown in Figure 6A, FoxR2 knockout significantly inhibited the phosphorylation of Akt and increased the expression levels of p27. In contrast, overexpression of FoxR2 increased the phosphorylation level of Akt and decreased the level of p27.

Next, we further analyzed the subcellular location of p27. We found that there was a significant increase in nuclear p27 when knocking out FoxR2, while a reduction of cytoplasmic p27 was observed. In contrast, overexpression of FoxR2 induced a remarkable reduction of nuclear p27 (Figure 6B–6D). These results suggest that FoxR2 may promote glioma cell proliferation, migration and invasion through decreasing the expression and nuclear location of p27.

## DISCUSSION

The Fox gene family play important roles in the development and progression of human cancers [3, 24]. FoxR2, belonging to the FoxR family, is a transcription factor [8]. It has been demonstrated that FoxR2 involoved in the tumorgenesis of human solid tumors, such as malignant peripheral nerve sheath tumors [9], medulloblastoma [10], breast cancer [12] and human hepatocellular carcinoma [13]. However, the functions of FoxR2 in human gliomas remain unclear. In this study, our results demonstrate that FoxR2 contributes to glioma cell proliferation, migration and invasion by regulating the expression of p27 and MMP-2.

Consistent with previous reports, our data show that the mRNA and protein level of FoxR2 are upregulated in glioma clinical samples [12, 13]. Tumor heterogeneity poses a major challenge to cancer treatment. Recently, it has been demonstrated that glioma intratumoral heterogeneity may underlie the inability of conventional and targeted therapies [25–27]. Consistent with these results, we found that there is a surviving sub-population resistance to FoxR2-knockout treatment. It is well known that the cyclin D1-CDK4 complex and the cyclin E-CDK2 complex hyperphosphorylate pRb during the G1-S transition [28]. We found that FoxR2 expression modulates multiple cell cycle regulatory gene (cyclin D1, cyclin E and p-Rb). Our data suggest that FoxR2 promotes glioma cell proliferation by regulating cell cycle progression.

Furthermore, we found that FoxR2 could accelerate glioma cell migration and invasion. Cell migration and invasion are dynamic processes with many different molecules [29]. Glioma cells migrate into the surrounding brain parenchyma though degradation of ECM by MMPs [15, 30]. MMP-2 is highly expressed in multiple cancers including gliomas, and is associated with tumor invasion [31, 32]. We found that FoxR2 promotes invasion and migration of glioma cells by increasing the expression and activity of MMP-2.

The elevated expression of p27 leads to G1 arrest and inhibits cell proliferation in many cancer cell types [33, 34]. The low expression of p27 is associated with high aggressiveness and poor prognosis in many tumor types [35, 36]. Furthermore, p27 has dual functions depending on its subcellular localization [18, 37]. Nuclear localization of p27 regulates the cell cycle progression from G_1_ to S-phase [17]. However, p27 phosphorylation by Akt impairs its nuclear import, resulting in its cytoplasmic localization [38]. Cytoplasmic p27 functions as an oncogene and mainly involves in cell motility and invasion [22, 37]. We found that FoxR2 knockout can decrease the level of p-Akt and reduce the cytoplasmic localization of p27. Our results are correlated with the roles of FoxR2 in glioma cell proliferation, migration and invasion. These data suggest that FoxR2 promotes the tumorigenicity of glioma through decreasing the expression and nuclear location of p27.

In summary, our results show that FoxR2 has critical roles in cell proliferation, migration and invasion of glioma. Importantly, overexpression of FoxR2 promotes glioma cell proliferation and invasion through decreasing p27 expression and increasing MMP-2 expression. These data indicate that upregulation of FoxR2 may confer enhanced tumorigenicity in glioma cells.

## MATERIALS AND METHODS

### Clinical tissue specimens

A total of 42 fresh specimens including 33 glioma tissues (obtained through surgical resection) and 9 non-tumorous brain tissues (internal decompression in cerebral trauma) were obtained from the Affiliated Hospital of Xuzhou Medical University (xuzhou, China). All the glioma specimens were histologically diagnosed based on World Health Organization (WHO) criteria. This study was approved by the ethics committee of the Affiliated Hospital of Xuzhou Medical University.

### Cell lines, antibodies and reagents

Cell lines including 293T, U87, U251, A172 and U118 were obtained from the Shanghai Cell Bank, Type Culture Collection Committee, Chinese Academy of Sciences. All of the cells were grown in DMEM supplemented with 10% fetal bovine serum (FBS) and incubated at 37°C in a humidified incubator with 5% CO_2_. Primary antibodies against FoxR2 and β-actin were obtained from Abcam (Cambridge, Massachusetts, USA). The antibodies against cyclin D1, p27, p21, cyclin E, p-Rb, Akt, p-Akt, MMP-2 and Histone H3 were purchased from Cell Signaling Technology (CST, Beverly, MA). DAPI was obtained from Sigma-Aldrich Chemical Co. (St. Louis, MO, USA).

### Construction and production of the lentivirus

Lentiviral CRISPR/Cas9 system was used to knock out FoxR2 in glioma cells. We designed two sgRNAs (FoxR2-sgRNA1 and FoxR2-sgRNA2) targeting human FoxR2 gene. The sgRNAs was synthesized and subcloned into the lentiCRISPRv2 transfer plasmid. For overexpression of FoxR2, the cDNA encoding human FoxR2 gene was inserted into the pWPXLd-puro lentiviral vector using *Bam*H I and *Mlu* I sites. The sequences of the primers used in this study are as follows: Control-sgRNA-F: 5′-CACCGTACTAACGCCGCTCCTACAG-3′; Control-sgRNA-R: 5′-AAACCTGTAGGAGCGGCGTTAG TAC-3′; FoxR2-sgRNA1-F: 5′-CACCGCATTACTCCGA CTCTGTATT-3′; FoxR2-sgRNA1-R: 5′-AAACAATAC AGAGTCGGAGTAATGC-3′; FoxR2-sgRNA2-F:5′-CAC CGCCCAATATCCTGTGCCCCCT-3′; FoxR2- sgRNA2-R: 5′-AAACAGGGGGCAGGATATTGGGC-3′; pWPXLd-FoxR2-F: 5′-CGGGATCCATGGACTTAA AACTAAAAG-3′; pWPXLd-FoxR2-R: 5′-CGACGCGT CCAAGATCAAAGAGAGAGGTCAAC-3′. The viruses were propagated in 293T cells by co-transfecting the corresponding plasmids with the helper plasmids pSPXA2 and pMD2.G. After 72 h incubation, the supernatant was collected and concentrated by ultracentrifugation.

### Establishment of stable cell lines

Glioma cell lines were transfected with the FoxR2-sgRNA1, FoxR2-sgRNA2, GFP-FoxR2, and corresponding control lentivirus for 72 h. Next, the cells were continuously cultured in the medium containing 2.5 μg/mL puromycin. The surviving cells were cultured and used to generate stable cell lines for following experiments.

### Cell viability assay

The cell viability was assessed via cell counting kit-8 assay (CCK-8, Dojindo, Janpa). Cells were plated at 3000 per well in triplicate in 96-well plates and incubated for 24 h, 48 h, 72 h and 96 h, respectively. CCK-8 reagent was added into each well and cultured for an additional 2 h. The absorbance was measured using a microplate reader at a wavelength of 450 nm.

### EdU incorporation assay

Cell proliferation was measured using an EdU assay kit (Ribobio, Guangzhou, China). Cells were seeded into 24-well plates and incubated for 24 h. Subsequently, the cells were treated with 50 μM EdU for 2 h and then fixed in 4% paraformaldehyde for 30 min. Subsequently, cells were permeabilized with 0.5% Triton X-100 and incubated with Apollo^®^ reaction cocktail for 30 min.Finally, cellular DNA was stained with DAPI for 15 min. The images were taken by an inverted microscope (Olympus, Japan).

### Colony formation assay

The cells were seeded at a density of 500 cells/well in 6-well culture plates. After a 14-day incubation, the cells were fixed in methanol for 15 min and stained with 0.1% crystal violet solution. After washing twice with PBS, the plates were photographed using a digital camera. Positive colony formation, defined as colonies with more than 50 cells, was confirmed by manual counting.

### Cell cycle assay

The cells were plated at a density of 2 ×10^5^ cells/well in 6-well plates. After incubation for 24 h, cells were collected and fixed in 70% ethanol for overnight at −20°C. Subsequently, the cells were stained with solution containing 50 μg/mL propidium iodide and 1 mg/mL RNase for 15 minutes. Finally, the cells were assayed for cell cycle distribution using a flow cytometer (FACSCalibur, Becton-Dickinson) and analyzed by CellQuest Pro software (BectonDickinson).

### Wound migration assay

The migration function of cells was evaluated using wound healing assay. The cells were seeded in 6-well plates and allowed to attach overnight. A rectangular lesion was created using a plastic pipette tip. The cells were washed with PBS to remove the debris and incubated in serum-free media for 36 h. At the designated time, five randomly selected fields at the lesion border were acquired under an inverted microscope (Olympus). The number of cells across the wound was normalized to the control group.

### Transwell invasion assay

The cell invasion assay was performed using a transwell system (Corning, NY) according to the manufacturer's protocol. Briefly, matrigel was diluted and applied to the top side of the 8-μm pore polycarbonate filter. Cells were resuspended in serum-free medium and added to the top chamber. In the lower chamber, the DMEM media containing 10% FBS was added. After 36 h of incubation, the noninvasive cells in the upper surface of the membrane were carefully removed, and the invading cells were fixed with 4% formaldehyde for 30 min and stained with a 0.3% crystal violet solution for 30 min. Five randomly selected fields of invading cells in each well were photographed using an inverted microscope and counted.

### Gelatin zymography assay

The activity of MMP-2 was examined by gelatin zymography assay. The cells (5 × 10^5^) were seeded in 12-well plates and incubated overnight at 37°C. The cells were washed twice with PBS and cultured for an additional 24 h in serum-free medium. Then, the media was harvested, centrifuged and resuspended in SDS loading buffer without β-mercaptoethanol. All samples were subjected to 10% SDS-PAGE (containing 0.2% gelatin) electrophoresis analysis. Gels were washed in 2.5% Triton X-100 for three times to remove SDS and then incubated overnight in reaction buffer. Subsequently, gels were stained with 0.25% Coomassie Brilliant blue R-250 and destained with 40% methanol and 10% acetic acid. The gelatinolytic activity of MMP-2 in the gel was detected as clear white bands on a dark background.

### Real-time RT-PCR analysis

The total RNA from non-tumorous brain tissues or glioma samples were isolated using the Trizol reagent (Invitrogen). Reverse transcription was performed by Transcriptor First Strand cDNA Synthesis Kit (Roche) according to according to the manufacturer's protocols. The mRNA level of FoxR2 in glioma tissues was detected by a TaqMan-based real-time PCR. The specific primers and TaqMan probes for FoxR2 and GAPDH were purchased from Life Technologies (Gaithersburg, MD). GAPDH gene of human was used as internal control for normalizing the expression level of FoxR2.

### Western blot analysis

The total, cytoplasmic or nuclear protein were used in Western blot analysis. The nuclear and cytoplasmic extraction kit (Pierce, Rockford, IL) according to the manufacturer's instructions. Equal amounts of protein (50 μg/lane) were separated by SDS-PAGE electrophoresis and transferred onto Polyvinylidene fluoride membrane (Roche). After blocking with 5% non-fat milk, the membrane was probed with special primary antibodies at 4°C overnight and secondary antibodies at room temperature for 2 h. The signals were detected using the enhanced chemiluminescence detection system.

cStatistical analyses were performed using the SPSS Version 16.0. The data were expressed as the mean ± SEM of three independent experiments. Comparisons of the mean values between the control and treated groups were performed using Student's *t* test. *P* values < 0.05 was considered as statistically significant.
